# Population Immunity to Influenza Neuraminidase: From Immune Imprinting to Pandemic Preparedness

**DOI:** 10.3390/vaccines14090748

**Published:** 2026-08-28

**Authors:** Kong Yen Liew, Tri Komala Sari, Yee-Joo Tan

**Affiliations:** 1 Infectious Diseases Translational Research Programme and Department of Microbiology and Immunology, Yong Loo Lin School of Medicine, National University of Singapore, Singapore 117545, Singapore; kongyen@nus.edu.sg (K.Y.L.); tri.komalasari@nus.edu.sg (T.K.S.); 2 Laboratory of Virology, Faculty of Veterinary Medicine, Udayana University, Denpasar 80234, Indonesia

**Keywords:** influenza, neuraminidase, neuraminidase-inhibiting antibodies, neuraminidase-binding antibodies, population immunity, immune imprinting, cross-reactivity, zoonotic influenza, pandemic preparedness

## Abstract

Neuraminidase (NA) is a major surface glycoprotein of influenza viruses that mediates the release of progeny virions from infected cells. Although both hemagglutinin (HA) and NA are targets of protective immune responses, current seasonal influenza vaccines are primarily standardized based on HA content, resulting in variable and often suboptimal induction of NA-specific immunity. Therefore, NA is subject to different immune pressures and exhibits distinct patterns of antigenic evolution. Increasing evidence suggests that neuraminidase-inhibiting (NAI) antibodies contribute to protection against influenza by limiting viral replication and reducing disease severity, particularly when HA antibodies are poorly matched to circulating strains. The recent emergence of highly pathogenic avian influenza (HPAI) H5N1 viruses with widespread transmission in dairy cattle and increasing zoonotic infections has renewed interest in understanding the extent of pre-existing human immunity to NA and its potential role in mitigating disease caused by emerging influenza viruses. However, the breadth of cross-reactive NA immunity against zoonotic influenza viruses remains incompletely understood. In this review, we summarize current knowledge of population immunity to influenza NA, with a focus on the roles of immune imprinting, natural infection, and seasonal vaccination in shaping NA-specific antibody responses. We further discuss the cross-reactivity of these antibodies against zoonotic influenza viruses and highlight the implications of NA immunity for pandemic preparedness.

## 1. Introduction

Influenza A and B viruses are a major global public health threat, causing an estimated 3 to 5 million cases of severe illness and 290,000 to 650,000 deaths each year [1]. Hemagglutinin (HA) and neuraminidase (NA) are two important surface antigens of influenza viruses, which mediate viral entry and release of progeny virions, respectively. Influenza A viruses can be classified according to their HA (H1–H18) and NA (N1–N11) subtypes [2]. Currently, H1N1 and H3N2 viruses co-circulate in humans and are responsible for annual epidemics.

The continuous evolution of influenza viruses under host immune pressure drives antigenic changes in both HA and NA. However, as current seasonal influenza vaccines are standardized based on HA content and primarily elicit HA-specific antibody responses, antigenic evolution of NA has received comparatively less attention [3]. Unlike HA, NA generally evolves more slowly and retains greater antigenic conservation within a subtype, making it an appealing target for broad protective immunity [3,4,5].

Increasing evidence indicates that NA antibodies represent an independent correlate of protection, associated with shortened symptom duration and reduced disease severity [6,7,8]. In contrast to HA antibodies, which primarily block viral entry, NA antibodies act predominantly after infection has been established. By inhibiting NA enzymatic activity, NA-inhibiting (NAI) antibodies restrict the release of progeny virions from infected cells, thereby preventing the initiation of new infection cycles [9]. In addition, by inhibiting NA activity, anti-NA antibodies are expected to preserve sialylated decoy receptors on airway mucins, thereby reducing viral penetration through the mucus layer and restricting access to the underlying epithelial cells [10]. Beyond direct inhibition of NA activity, NA-specific antibodies can also promote viral clearance through Fc-mediated effector functions, including antibody-dependent cellular cytotoxicity (ADCC) and antibody-dependent cellular phagocytosis (ADCP) [11,12,13]. Collectively, these mechanisms reduce viral replication and disease severity, complementing HA-mediated immunity that primarily prevents initial infection.

Although this review primarily focuses on antibody-mediated NA immunity, NA also contains T-cell epitopes recognized by CD4^+^ and CD8^+^ T cells [14,15]. NA-specific T-cell responses have been demonstrated experimentally and may contribute to protection against influenza. In an APC-targeted DNA vaccination study, NA immunization induced both NA-specific antibodies and T-cell responses, with protection being primarily mediated by NAI antibodies [16]. However, depletion of T cells significantly reduced protection when anti-NA antibody levels were low, indicating that NA-specific T-cell responses may confer protection [16]. Nevertheless, the contribution of NA-specific T-cell responses to influenza immunity in humans remains poorly characterized. Hence, the subsequent sections will focus on B-cell-mediated immunity, specifically NA-binding and NA-inhibiting antibodies.

Despite growing recognition of the protective role of NA antibodies, population immunity to influenza NA remains less well characterized than immunity to HA. Most influenza surveillance and vaccine effectiveness studies focus primarily on HA-specific antibody responses, while NA-specific antibodies are not routinely measured [17]. Consequently, there is limited understanding of how repeated influenza infections and seasonal vaccination shape population-level NA immunity across different age groups and birth cohorts. This knowledge gap has become increasingly important as emerging zoonotic influenza viruses continue to acquire the capacity to infect humans.

The unprecedented global spread of clade 2.3.4.4b H5N1 viruses among wild birds, poultry, dairy cattle, and other mammalian hosts, together with increasing zoonotic infections [18], has renewed interest in the potential contribution of pre-existing NA immunity to protection against emerging influenza viruses. Influenza A NAs are phylogenetically classified into Group 1 (N1, N4, N5, and N8) and Group 2 (N2, N3, N6, N7, and N9) [19]. More recently, the N10 and N11 subtypes identified in bat H17N10 and H18N11 viruses were found to lack canonical NA activity and have been proposed as Group 3 [19,20,21]. The degree of protection conferred by pre-existing NA-specific antibodies against emerging zoonotic influenza viruses is expected to depend on the antigenic relatedness of their NAs to that of previously circulating seasonal influenza viruses. However, the breadth of cross-reactive NA immunity against emerging influenza viruses remains incompletely understood.

This review summarizes current knowledge of population immunity to influenza NA, focusing on how immune imprinting, natural infection, and seasonal vaccination shape NA-specific antibody responses. We further discuss the role of cross-reactive NA immunity against zoonotic influenza viruses and highlight its implications for pandemic preparedness.

## 2. Literature Search

Relevant studies were identified through PubMed, Scopus, and Web of Science using the search terms “influenza” AND “neuraminidase” AND “antibody”. No restrictions were placed on the study period, and the last search was conducted on 21 August 2026. Eligible articles included original human studies reporting NA antibody levels, whether pre-existing levels in healthy individuals or measured before and after vaccination or infection. Extracted information included participant age, vaccination or infection status, influenza subtype, and methods used for NA antibody measurement. Review articles and animal studies were excluded. It is important to note that a systematic review has previously evaluated the efficacy of seasonal influenza vaccination in inducing NAI antibody titers against seasonal influenza A and B virus strains [22]. To avoid overlap in scope, the present review focuses primarily on the cross-reactivity of NA-specific antibodies against zoonotic influenza strains. Studies measuring NA antibody responses to seasonal influenza are described narratively in the text but not included in the summary table.

## 3. Development of Population NA Immunity

Antibody responses to influenza viruses are established through repeated exposure and shaped by an individual’s immune history [23]. Similar to HA, the initial encounter of the influenza virus during childhood can establish long-lasting immunological memory that influences antibody responses to subsequent influenza virus exposures, a phenomenon known as immune imprinting (see review by [23]).

**Figure 1:**
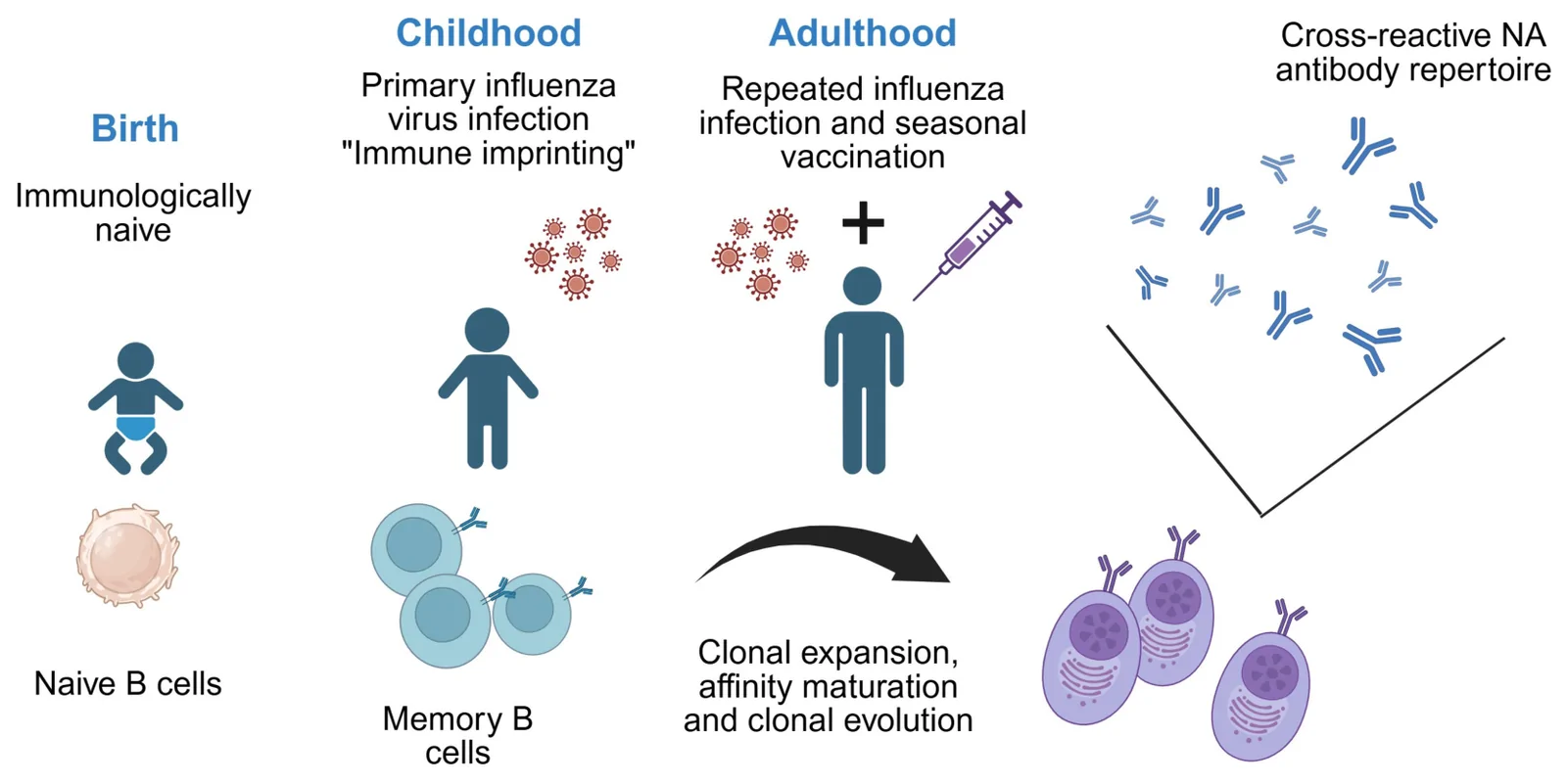
Lifelong development of population NA immunity. Individuals are born immunologically naive with a repertoire of naive B cells. Primary influenza virus infection during childhood establishes NA-specific memory B cells through immune imprinting. Subsequent influenza infections and seasonal vaccination recall these pre-existing memory B cells, driving affinity maturation and clonal evolution that progressively broaden the NA-reactive antibody repertoire. Created in BioRender.

Unlike HA, NA undergoes slower antigenic drift, and thus anti-NA antibodies may provide protection even when HA is mismatched [4,5]. However, as NA is less abundant than HA on influenza virions and less immunodominant, NA-specific antibody responses are generally lower than HA-specific responses following natural infection or vaccination [23]. Despite this, anti-NA antibodies are increasingly recognized as an independent correlate of protection against influenza, being associated with reduced viral replication, shorter symptom duration, and decreased disease severity [6,7,8]. Recent studies have further demonstrated that healthy individuals harbor broadly reactive NA-specific memory B cells capable of recognizing conserved epitopes shared among diverse influenza viruses [24,25]. These pre-existing memory B cells can be recalled by subsequent influenza infection or vaccination and undergo further clonal expansion and affinity maturation to generate broadly reactive antibodies [25]. These findings suggest that broad NA reactivity can be acquired through clonal evolution following repeated influenza exposures, providing a mechanistic basis for how immune imprinting shapes population NA immunity [24,25] (Figure 1).

**Figure 2:**
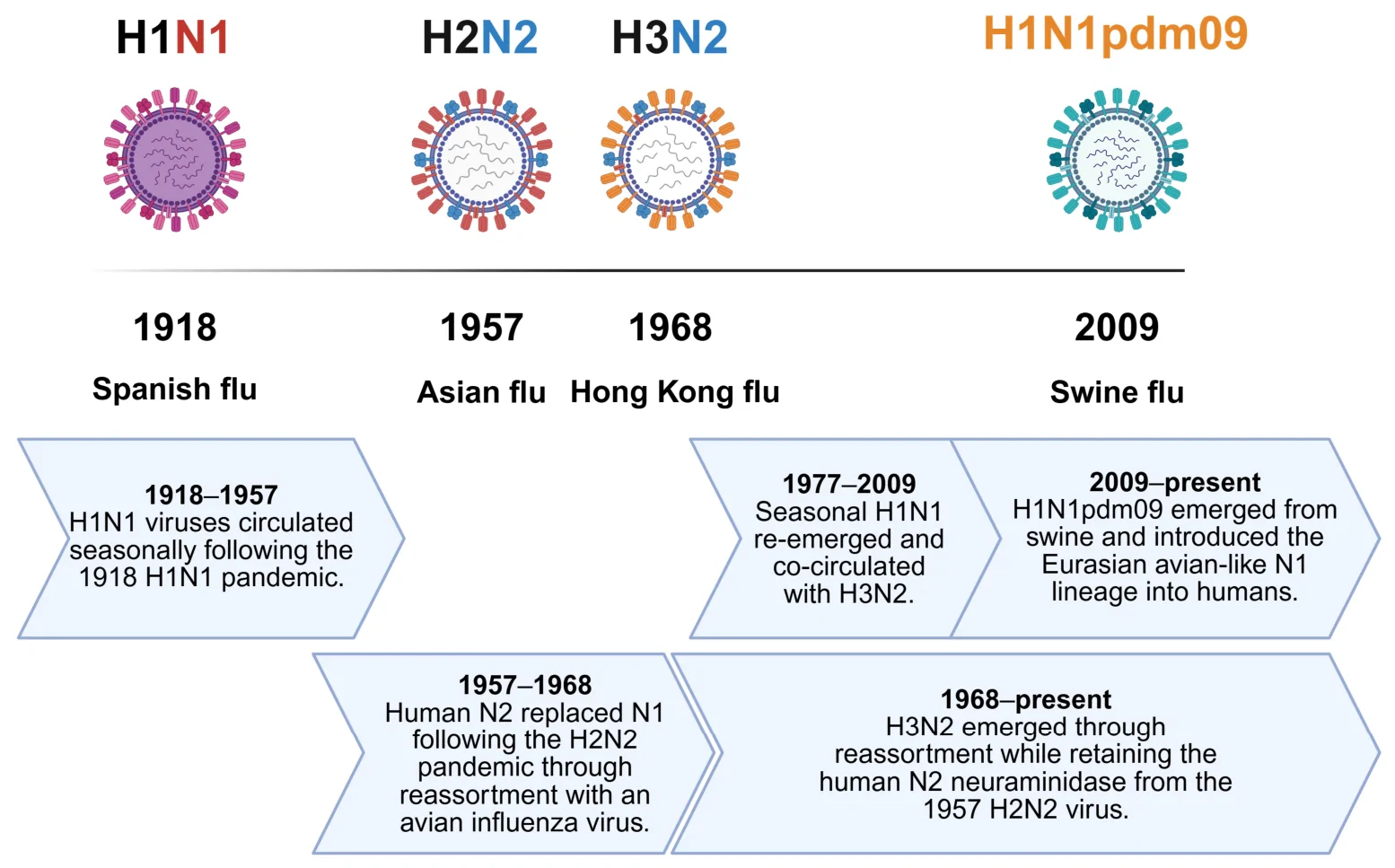
Historical circulation of influenza A virus NA subtypes in humans and their implications for population immunity. The 1918 H1N1 pandemic introduced N1 into the human population, followed by replacement with N2 during the 1957 H2N2 pandemic. The 1968 H3N2 pandemic retained the human N2 NA through reassortment, while seasonal H1N1 re-emerged in 1977 and co-circulated with H3N2 until the emergence of the 2009 pandemic H1N1 (H1N1pdm09) virus, which replaced the previous seasonal H1N1 lineage. These sequential changes in N1 and N2 circulation established distinct birth cohort-specific exposure histories, shaping immune imprinting and population-level NA immunity. Adapted from [31] and created in BioRender.

The historical circulation of influenza A viruses has resulted in distinct birth cohorts with different NA immunity profiles. Individuals born between 1957 and 1968 were first exposed to the H2N2 pandemic virus, which introduced N2 into the human population [26] (Figure 2). The subsequent reassortment of the human H2N2 virus with an avian H3 virus generated the novel H3N2 virus that caused the 1968 Hong Kong influenza pandemic [26]. Interestingly, higher levels of NAI antibodies were later found to be associated with decreased infection risk during the 1968 pandemic [27]. This suggests that anti-N2 antibodies acquired from prior H2N2 exposure between 1957 and 1968 contributed to protection despite the population being immunologically naive to the new H3 hemagglutinin. Remarkedly, recent studies show that NAI antibodies against A/Hong Kong/1/1968 (H3N2) or A/Achi/2/1968 (H3N2) remain elevated in individuals born during this period [28,29,30], likely maintained through repeated exposures to drifted H3N2 strains throughout their lives.

Consistently, recent serological studies have demonstrated clear birth cohort effects in N1 NAI antibody responses. Multiple serological studies have consistently shown the highest N1 NAI titers in individuals born during the late 1990s and early 2000s [29,30,32,33], who experienced the 2009 pandemic and repeated exposure through seasonal infection during childhood and adolescence. Interestingly, one study also reported that anti-N1 titers showed a secondary peak among the oldest individuals born during the pre-1947 H1N1 era [29]. These findings suggest that immune imprinting contributes substantially to population heterogeneity in NA immunity. This concept is also supported by an earlier serological study demonstrating evidence of original antigenic sin, in which NA antibody responses (both NA-binding and NAI antibodies) remained preferentially directed toward NA antigens encountered during childhood despite subsequent exposure to antigenically drifted influenza viruses [34]. Consistent with this, influenza virus infection boosts NAI antibodies in an age- and subtype-dependent manner, reflecting an individual’s cumulative exposure history rather than the infecting virus alone [35].

Taken together, population NA immunity reflects a combination of immune imprinting and lifelong antigenic exposure. Primary infection establishes NA-specific memory B cells that are subsequently recalled and refined through repeated influenza infections and vaccination, resulting in progressively broader and more durable antibody responses. Sequential exposure to antigenically drifted viruses carrying the same NA subtype further promotes the expansion of cross-reactive antibodies while maintaining recognition of conserved NA epitopes. Consequently, distinct birth cohorts develop unique NA immunity landscapes that influence susceptibility to seasonal influenza and the level of pre-existing immunity against emerging influenza viruses. Understanding cohort-specific differences is therefore essential for interpreting age-dependent susceptibility to seasonal influenza and forms the basis for understanding how seasonal vaccination further shapes population NA immunity.

## 4. Impact of Seasonal Influenza Vaccination on NA Immunity

In addition to natural infection, vaccination is another primary driver of NA-specific antibodies in human populations [36]. Seasonal influenza vaccines are currently available as inactivated influenza vaccines (IIVs), including split-virus and subunit formulations, live attenuated influenza vaccines (LAIVs), recombinant HA vaccines (RIVs), and enhanced formulations such as adjuvanted and high-dose vaccines for older adults [37]. However, unlike HA, which is standardized in all licensed influenza vaccines, the amount, integrity, and enzymatic activity of NA are not regulated during vaccine production. Consequently, the capacity of different vaccines to induce NA-specific antibody responses varies considerably [36,38].

Although both infection and vaccination induce NA-specific antibodies, accumulating evidence suggests that natural infection generally elicits stronger and broader NAI antibody responses than current seasonal influenza vaccines [39,40,41]. During infection, the immune system encounters native tetrameric NA expressed on both the virions and infected cells, promoting robust activation of NA-specific B cells. Accordingly, influenza virus infection has been shown to induce a higher proportion of NA-reactive B-cells than vaccination, likely due to exposure to intact viral epitopes that are not well preserved in commercially available inactivated influenza vaccines [39].

Among licensed vaccines, most evidence has been generated for IIVs. As these vaccines are standardized according to HA content rather than NA, manufacturing processes may compromise NA stability, resulting in substantial variation in NA antigen content between vaccine manufacturers [42,43]. Consequently, studies evaluating vaccine-induced NAI antibody responses have reported considerable variability. Nevertheless, a recent systematic review reported that split-virus vaccines can boost NAI titers against vaccine-matched N1, N2, and influenza B NA, although considerable variability was observed among different commercial vaccines [22].

Although LAIVs, including FluMist and Fluenz Tetra, expose recipients to native NA, they generally induce weak serum NAI antibody responses [8,22,44,45,46,47,48]. A comparative study evaluating six licensed influenza vaccines (five IIVs and one LAIV) found that FluMist elicited the weakest NAI antibody responses despite containing native NA [44]. Several factors likely contribute to this limited immunogenicity. First, as LAIVs are administered intranasally, they primarily induce mucosal IgA and cellular immune responses rather than systemic antibody responses [45,49]. Besides that, in adults with pre-existing influenza immunity, replication of the attenuated vaccine virus in the upper respiratory tract may be restricted by high levels of pre-existing antibodies, limiting viral antigen production and reducing the amount of NA available to stimulate memory B cells [44,49].

Two distinct LAIV backbones are currently in use, which are the Ann Arbor backbone (FluMist and Fluenz Tetra, AstraZeneca, Cambridge, England) and the Russian backbone (Ultravac, NPO Microgen, Irkutsk, Russia; Nasovac-S, Serum Institute of India, Pune, India) [50]. Evidence suggests that weak systemic NAI responses are not universal across both platforms. Russian backbone LAIVs, which are based on the cold-adapted A/Leningrad/134/17/57 (H2N2) master donor virus, have been shown to induce NAI antibody responses against both seasonal and pandemic influenza viruses [51]. In a study evaluating several Russian backbone LAIVs, LAIVs targeting H1N1pdm09, H5N2, H2N2, and H7N3 produced at least a twofold increase in NAI titers in 6–29.6% of vaccinees in the absence of concomitant hemagglutination inhibition (HI)/microneutralization (MN) seroconversion [51]. By contrast, LAIV targeting H7N9 failed to induce NAI seroconversion without concurrent HA antibody induction, underscoring the immunodominance of HA over N9 [51]. Besides that, vaccination with seasonal trivalent LAIV (2005–2006 season) containing A/New Caledonia/20/99 (H1N1) boosted NAI antibodies against homologous H1N1 as well as heterologous A/California/7/09 (H1N1pdm09) and A/Vietnam/1203/04 (H5N1) (Table 1) [51]. While Russian backbone LAIVs effectively induced systemic IgG against H3 HA and influenza B, reduced immunogenicity was observed for the H1N1 component [50,52,53]. However, as these studies did not directly assess NA antibodies, it remains unclear whether the observed strain-specific differences extend to NA. Hence, LAIV-induced NAI responses may be influenced by the vaccine backbone, vaccine strain, and pre-existing immunity.

Although split virus IIVs can increase NAI antibody titers, the magnitude and durability of these responses vary considerably between individuals [22]. Longitudinal studies have shown that vaccine-induced NAI antibody titers gradually decline following seasonal influenza vaccination [54,55], although the persistence of these responses remains less well characterized than that of HA-specific antibodies. Vaccine responsiveness is also strongly influenced by pre-existing NA immunity. Individuals with low baseline NAI titers generally exhibit greater relative increases following vaccination, whereas those with high baseline titers often demonstrate smaller fold rises [22].

Despite the relatively slow antigenic evolution of NA compared with HA, amino acid substitutions continue to accumulate within the NA head domain of circulating seasonal N1 and N2 viruses and can alter antibody recognition [56,57,58]. These observations highlight the need for continued antigenic surveillance and have important implications for the development of next-generation NA-based vaccines. In addition, both adjuvants and routes of administration have also been shown to influence NA immunogenicity, indicating that optimal boosting of NAI antibody responses requires carefully designed formulations tailored to the delivery method [59,60,61,62].

Overall, seasonal influenza vaccination contributes to maintaining population NA immunity but remains less effective than natural infection in inducing broad and durable NAI antibody responses. This distinction has important implications for population immunity, as differences in infection history and vaccination practices influence the magnitude and breadth of NAI antibodies across different age groups. Understanding how these factors shape long-term NA immunity will be critical for optimizing future vaccines and assessing their potential to enhance protection against both seasonal and emerging zoonotic influenza viruses.

**Table 1:** Studies on human NA antibodies with cross-reactivity against zoonotic influenza viruses.

References	Study Population	Vaccination/Infection Status	Assay Used	NA Tested	Key Findings
Sandbulte et al. (2007) [63]	Healthy (*n* = 38)	Unknown	NA inhibition test [64]	H1N1 (A/New Caledonia/20/99) H5N1 (A/Hong Kong/213/03 and A/Vietnam/1203/03)	- Notably, 7 of 38 individuals had at least 1:20 cross-reactive NAI antibodies against both H5N1 viruses (A/Hong Kong/213/03 and A/Vietnam/1203/03).
Changsom et al. (2017) [65]	H5N1 survivors (*n* = 4, 2–32 years) and H1N1pdm patients (*n* = 20, 18–42 years)	H5N1 survivors (Infected with clade 1 H5N1 virus in 2004 and 2005); H1N1pdm patients (Natural infection in 2011)	ELLA (NAI)	H1N1 (A/Puerto Rico/8/1934 and A/Thailand/104/2009) H5N1 (A/Thailand/1(KAN-1)/04 and A/Laos/Nong Khai 1/2007)	- H5N1 survivors had cross-reactive NAI antibodies against both H5N1 (KAN-1 and NK-1) and H1N1 (pdm and PR8) NAs. - H1N1pdm patients had cross-reactive NAI antibodies against H1N1pdm, H1N1-PR8 and H5N1-KAN-1 NAs.
Desheva et al. (2020) [51]	Retrospective analysis of sera in 2007, 2009, 2012, 2013 and 2014 Healthy sera (2005, 18–20 years), archived sera (2010–2011) and patient sera with laboratory-confirmed influenza A (2016)	Vaccination with seasonal trivalent LAIV (2005–2006 influenza season) containing A/17/New Caledonia/99/145 (H1N1), A/17/California/04/06 (H3N2) and B/60/Jilin/01/1 (B/Yamagata-like)	ELLA (NAI)	A/New Caledonia/20/99 (H1N1) A/California/7/09 (H1N1) A/South Africa/3626/2013 (H1N1) A/Vietnam/1203/04 (H5N1) A/California/1/1966 (H2N2) A/Leningrad/134/17/57 (H2N2) A/tern/South Africa/61 (H5N3) A/Anui/1/2013 (H7N9)	- Cross-reactive NAI antibodies against A/Vietnam/1203/04 (H5N1) increased from 16% in 2005 to 46.2% in 2011. - Pre-existing NAI antibodies against A/Anhui/1/13 (H7N9) were not detected. - Notably, 16.4% had a pre-existing NAI titer of ≥1:20 against A/mallard/NL/12/00 (H7N3). - HI and NAI antibody titers to A/California/7/09 (H1N1pdm09) influenza virus after natural infection were higher compared with LAIV vaccination.
Daulagala et al. (2024) [66]	Healthy sera 2009 (*n* = 50, 17–55 years) vs. sera 2020 (*n* = 63, 18–73 years)	Unknown	ELLA (NAI)	H1N1 (A/California/04/2009) H5N1 (A/black-faced spoonbill/Hong Kong/AFCD-HKU-22-21429-01012/2022)	- Overall, 96.8% (61/63) of 2020 sera had cross-reactive NAI antibodies to H5N1 NA compared with 42% (21/50) of 2009 sera. - Highest N1 NAI titers in individuals aged 10–19 and 20–29.
Liang et al. (2024) [28]	Healthy (EPI-HK cohort, *n* = 120, 1–82 years; Guangzhou cohort, *n* = 112, 6–83 years); Infected (CARES cohort, *n* = 43, 60–89 years)	Healthy (EPI-HK and Guangzhou cohorts, unknown); H3N2 infection (CARES cohort, 2015–2017 influenza seasons)	ELLA (NAI)	H3N2 NAs between 1968 and 2016 (*n* = 11) H9N2 NAs between 1997 and 2015 (*n* = 8)	- NAI titers against H9N2 increased with age and correlated with the 1968 H3N2-Aichi titers. - The 1968 H3N2-Aichi immune sera were protective against H9N2 challenge in mice. - 1957–1968 cohort: high titers to H3N2-Aichi, modest to avian N2s. Pre-1957 cohort: high titers to both H3N2-Aichi and avian N2s. - N3 and N9 NAI titers were highest among the elderly aged 60–69 years. - The majority of infected sera (84.1%) seroconverted to 2014 H3N2-HK NA, whereas cross-seroconversion to avian N2, N3, N5, N7, and N9 occurred at low rates (2.3–25.6%).
Li et al. (2025) [67]	Healthy (*n* = 489, 1–88 years); Vaccinated (*n* = 59, 0.5–92 years); Infected (H1N1, *n* = 62, 19–74 years; H3N2, *n* = 35, 18–77 years)	Seasonal IIV4 (2023–2024 influenza season); Natural infection (2023–2024 influenza season)	MIADA (NA-binding); ELLA (NAI)	H1N1 (A/Wisconsin/588/2019) H5N1 (A/American wigeon/South Carolina/22- 000345-001/2021 and A/Texas/37/2024)	- Cross-reactive N1-binding antibodies to H1N1 and H5N1 were detected in most individuals ≥ 18 years old. - H1N1pdm, but not H3N2 infection, boosted both NA-binding and NAI antibodies to H5N1 NA. - Seasonal vaccination boosted NAI antibodies, but not NA-binding antibodies to H5N1 NA.
Werner et al. (2025) [33]	Vaccinated (*n* = 50, 23–68 years); Infected (*n* = 23, mean age 49.87 years)	Seasonal IIV3 (2024–2025 influenza season); Natural infection (2023–2024 influenza season)	ELISA (NA-binding); ELLA (NAI)	H1N1 (A/California/4/2009) H5N1 (A/Bovine/Texas/24-029328-01/2024)	- At baseline, NA-binding and NAI antibody titers against H5N1 NA were comparable to those against H1N1 NA. - Seasonal vaccination did not increase NA-binding and NAI antibodies against H5N1 NA. - H1N1pdm, but not H3N2 infection, boosted both NA-binding and NAI antibodies to H5N1 NA. - NA-binding antibodies against both H1N1 and H5N1 NAs negatively correlated with age (individuals born around the 2000s have the highest titers).
Liew et al. (2026) [68]	Infected (2023–2025 influenza seasons, *n* = 34, 28–76 years)	Natural infection (2023–2025 influenza seasons)	ELLA (NAI)	H1N1 (A/California/4/2009 and A/Victoria/4897/2022) H5N1 (A/Texas/37/2024 and A/Bangladesh/Khulna/IEDCR-icddr,b-IC2/2025)	- H1N1pdm infection boosted cross-reactive NAI antibodies to clade 2.3.4.4b H5N1-Texas NA, but not after H3N2 infection. - Cross-reactive NAI antibodies showed reduced inhibition of clade 2.3.2.1a H5N1-Bangladesh NA.
Singh et al. (2026) [32]	Healthy (*n* = 300, 18–85 years)	Unknown	ELISA (NA-binding); ELLA (NAI)	H1N1 (A/California/4/2009) H5N1 (A/bald eagle/FL/W22-134-OP/2022, A/Vietnam/1203/2004, A/California/135/2024, A/Louisiana/12/2024 and A/British Columbia/PHL−2032/2024) H5N5 (A/great black-backed gull/NS/FAV-0405-1/2023 and A/Washington/2148/2025) H7N9 (A/Anhui/1/2013)	- NA-binding antibody titers ranked: H5N1 Louisiana > H1N1 Cal09 > H5N1 FL22 > H5N1 Cal24 > H5N1 VN04. - NAI antibody titers ranked: H5N1 Louisiana > H5N1 FL22 > H1N1 Cal09 > H5N1 Cal24 > H5N1 VN04. - NAI antibody titers against H5N1-British Columbia NA were lower compared to H5N1-Louisiana NA due to an additional N-linked glycosylation site. - NA-binding antibodies against N5 and N9 as well as NAI antibodies against N5 were minimal.
Nair et al. (2026) [69]	Healthy (*n* = 64)	Unknown	ELISA (NA-binding)	H1N1 (A/Puerto Rico/8/1938) H5N1 (A/bovine/Ohio/B24OSU-342/2024)	- All sera neutralized H1N1-PR8 while 66% (42/64) showed neutralizing activity against H5N1-Ohio. - Neutralization correlated with N1-binding titers, but not H5 HA (full-length or stem) binding titers. - Depletion of N1-binding antibodies markedly reduced H5N1 neutralization.
Skowronski et al. (2026) [29]	Healthy (*n* = 575, 1–80 years, 10 birth cohorts)	Unknown	ELLA (NAI)	H1N1 (A/Mexico/InDRE4487/2009 and A/Wisconsin/67/2022) H5N1 (A/RT-Hawk/ON/FAV-0473-4/2022) H3N2 (A/Hong Kong/01/1968 and A/Massachusetts/18/2022)	- In total, 404/575 (70%) had detectable H5N1 NAI titers. - NAI titers against H1N1pdm09 and H5N1 were highest among young adults born 1997 to 2003 and lowest among pediatric cohorts born 2015 to 2023 and middle-aged adults born 1957 to 1967. - A secondary peak of N1 NAI titers was observed among the oldest cohorts born during the pre-1947 H1N1 era. - NAI titers against 1968 H3N2 NA were all higher than 2022 H3N2 NA.
Ort et al. (2026) [30]	Healthy (sera from 2017, *n* = 120 adults born 1927–1999 and *n* = 36 children born 2000–2016; sera from 2005, *n* = 31 born 1918–1940 and *n* = 34 born 1942–1983); Infected (*n* = 25 born 2006–2023)	Healthy (Unknown); Natural infection (2023–2025 influenza seasons)	ELLA (NAI)	H1N1 (A/California/07/2009 and A/Wisconsin/50/2022) H5N1 (A/Vietnam/1203/2005, A/Dairy Cow/Texas/24− 008749−002−v/2024 and A/British Columbia/PHL−2032/2024) H3N2 (A/Thailand/8/2022) H5N5 (A/Washington/2148/2025)	- 2017 sera: elderly born 1918–1957 and young adults born around the 2000s had the highest NAI titers against H1N1 and both clade 1 and 2.3.4.4b H5N1 NAs. - Many children had undetectable or low levels of NAI antibodies. - H1N1, but not H3N2, elicited cross-reactive NAI antibodies against H5N1 NA in children. - 2005 sera: Individuals born 1918–1957 had cross-reactive NAI antibodies against both H1N1pdm09 and H5N1 NA. - Pre-1968 cohorts had the highest N2 NAI titers against 1968 H3N2 NA. Highest N2 NAI titers against 2009 H3N2 NA were detected in individuals born just before 2009. - Only 21% of 2017 sera (29/139) had an NAI titer of ≥40 against H5N5 NA.

Abbreviations—LAIV: live-attenuated influenza vaccine; IIV3/IIV4: trivalent and quadrivalent seasonal inactivated influenza vaccine; NAI: neuraminidase inhibition; MIADA: multiplex influenza antibody detection assay; ELISA: enzyme-linked immunosorbent assay; ELLA: enzyme-linked lectin assay.

## 5. Cross-Reactive NA Immunity to Zoonotic Influenza Viruses

### 5.1. Cross-Reactive Immunity Against Avian N1 (H5N1)

Highly pathogenic avian influenza (HPAI) H5N1 virus was first detected in China as the goose/Guangdong (gs/Gd) lineage, which subsequently diversified into multiple viral clades [70]. Since the first detection of clade 2.3.4.4b H5N1 viruses in Europe in 2020, they have spread globally, with outbreaks reported across Africa, the Middle East, and the Americas [70] and more recently detected in Australia and New Zealand. From 2024 to 2025, infections with clade 2.3.4.4b H5N1 viruses led to 70 confirmed human cases in the United States linked to exposure to infected cattle and poultry [18], raising a huge concern about pandemic potential.

Despite the limited direct exposure of human populations to avian influenza viruses, recent studies have demonstrated that healthy individuals across diverse age groups and geographic regions possess antibodies directed against the N1 NA of the contemporary clade 2.3.4.4b H5N1 viruses (Table 1). High titers of NAI antibodies to the NA of a clade 2.3.4.4b H5N1 virus (A/black-faced spoonbill/Hong Kong/AFCD-HKU-22-21429-01012/2022) were detected in sera collected from healthy adults in Hong Kong in 2020, but not sera collected in 2009, suggesting that the exposure to the 2009 H1N1 pandemic virus elicited cross-reactive NAI antibodies against avian H5N1 [66]. Subsequent studies using sera collected between 2021 and 2024 further confirmed the presence of pre-existing NAI antibodies against clade 2.3.4.4b H5N1 NA, which correlated with titers against H1N1pdm [29,32,33,67]. Individuals born around the 2000s appeared to have the highest antibody levels [29,32,33], whereas children born well after the 2009 pandemic exhibited the lowest levels of cross-reactive NA-binding and NAI antibodies, consistent with imprinting by H1N1 NA [30,32]. Evidence from infection cohorts further supports a crucial role for H1N1pdm infection in boosting cross-reactive immunity, as recent H1N1, but not H3N2, infection has been shown to induce cross-reactive NAI antibodies against clade 2.3.4.4b H5N1 NA [30,33,67,68]. Whether seasonal influenza vaccination can similarly boost H5N1 NA antibodies remains uncertain [33,67], as the NA content in current influenza vaccines is not standardized. Notably, two studies involving individuals born before 1957, during the era of seasonal H1N1 circulation, also showed that these very old cohorts possessed cross-reactive NAI antibodies against clade 2.3.4.4b H5N1 NAs [29,30]. Earlier studies further demonstrated cross-reactive NAI antibodies against clade 1 H5N1 viruses: Sandbulte et al. [63] reported that 7 of 38 healthy individuals had detectable NAI antibodies against clade 1 H5N1 (A/Hong Kong/213/03 and A/Vietnam/1203/03), while Changsom et al. [65] showed that H5N1 survivors and H1N1pdm patients possessed cross-reactive NAI antibodies to both clade 1 H5N1 and H1N1 NAs. More recent work indicates that cross-reactive NAI antibody titers against clade 1 H5N1 are generally lower than those against clade 2.3.4.4b [32], suggesting that patterns of cross-reactivity vary across time and viral clades. Together, these findings indicate that cross-reactive NA immunity extends across multiple H5N1 clades, with imprinting by H1N1pdm playing a central role in shaping responses to contemporary clade 2.3.4.4b viruses, while very old cohorts may also retain additional cross-reactivity from exposures predating 1957.

NA immunity represents an underappreciated component of population-level protection against emerging avian influenza viruses, particularly given that human populations generally possess low antibody responses against the H5 hemagglutinin [32,67]. While NAI antibodies are often considered the primary functional correlate, recent findings indicate that NA-binding antibodies can also contribute to neutralization. In one study, serum neutralizing activity correlated with NA-binding titers but not H5 HA-binding titers, and depletion of NA-binding antibodies significantly reduced neutralization of H5N1 virus [69]. Moreover, immunization of mice with N2 NA conferred heterologous influenza A protection even in the absence of cross-NA-inhibiting antibodies [71], suggesting that NA-binding antibodies may exert protective effects. This suggests that NA-binding antibodies, even without classical NAI activity, may play a protective role, consistent with monoclonal antibodies that mediate ADCC despite lacking NAI activity [11,12,13]. Supporting this, several independent groups have shown that both cross-reactive NA-binding [72,73,74] and NAI antibodies [73] against clade 2.3.4.4b H5N1 NA, elicited by infection with the 2009 H1N1 pandemic virus, attenuate viral replication and pathogenesis in ferret models.

While these findings highlight the importance of NA immunity, it is critical to recognize that H5N1 viruses comprise divergent genotypes with NAs that are antigenically distinct. Clade 2.3.4.4b H5N1 viruses of the D1.1 genotype are of greater concern, as they have been associated with more severe cases compared to those of the B3.13 genotype [75]. Notably, D1.1 genotype viruses carry a North American avian N1, whereas B3.13 genotype viruses carry a Eurasian avian N1 [75]. The D1.1 NA genes are highly divergent from those of the B3.13 genotype, with the North American avian N1 differing by 23 amino acids, 11 of which are located on the globular head domain [76]. Although NAI titers against the clade 2.3.4.4b D1.1 H5N1 strain in a healthy population were reported to be higher than those against the B3.13 genotype, markedly lower titers were observed against a D1.1 genotype A/British Columbia/PHL-2032/2024 (H5N1) virus that caused a severe case in British Columbia [32]. This strain carries an additional putative N-linked glycosylation site at position 270, suggesting that viruses with this glycosylation site may pose a higher risk of severe infection [32].

While clade 2.3.4.4b H5N1 is the dominant clade globally, other clades remain endemic in parts of Asia, including clade 2.3.2.1a in India, Bangladesh, and Nepal [77,78] and clade 2.3.2.1e in Cambodia [79]. Notably, clade 2.3.2.1a H5N1 has also been implicated in a human infection in an Australian traveler returning from India [80]. Importantly, H1N1pdm-derived cross-reactive NAI antibodies may not exhibit equivalent potency against all H5N1 clades. It has been shown that cross-reactive NAI antibodies show reduced inhibition of clade 2.3.2.1a NA compared with clade 2.3.4.4b, reflecting antigenic divergence of NAs between these two clades [68]. Besides that, a study in Nepal identified a reassortant H5N1 virus carrying four gene segments (HA, NA, NP, and M) from clade 2.3.2.1a and the remaining four (NS, PB1, PB2, and PA) originating from clade 2.3.4.4b [81]. Thus, the co-circulation of clade 2.3.4.4b and 2.3.2.1a may facilitate the emergence of novel reassortants with heightened zoonotic potential, particularly when pre-existing HA and NA antibody responses to clade 2.3.2.1a viruses are low.

### 5.2. Cross-Reactive Immunity Against Other Zoonotic NA Subtypes

In contrast to the widespread immunity observed against avian N1 NAs, cross-reactive antibodies against other avian NA subtypes appear considerably less prevalent. For example, studies have shown that NAI antibodies against the recent A/Washington/2148/2025 (H5N5), which caused a fatal human infection in the United States, are low to minimal despite some individuals exhibiting high NAI titers against H5N1 NAs, indicating that phylogenetic relatedness within Group 1 NA does not necessarily translate into antigenic cross-reactivity [30,32].

Nevertheless, historical circulation of human H2N2 and H3N2 viruses has generated long-lived immunity to N2 NA that may contribute to cross-reactive antibody responses against avian H9N2 viruses. A study by Liang et al. [28] demonstrated that individuals born before 1957 have high pre-existing NA antibodies against the historical A/Singapore/1/1957 (H2N2) and A/Aichi/2/1968 (H3N2) NAs, which also cross-reacted with avian H9N2 NAs [28]. Notably, individuals aged 60–69 and 70–79 exhibited higher NAI titers to avian N3, N5, N7, and N9 compared with younger adults aged 21 to 39 [28]. These findings further support the concept that immune imprinting shapes the breadth of NAI responses.

Although H9N2 viruses generally cause mild disease in humans, they are enzootic throughout poultry populations in Asia and have donated their internal gene segments to several zoonotic influenza viruses, including H7N9, H10N8, and H3N8 [82]. Consequently, understanding population immunity to H9N2 NA is important not only for H9N2 infections but also for assessing immunity to future reassortant viruses.

In contrast, evidence for naturally acquired cross-reactive NA antibodies against H7N9 viruses remains limited. Pre-existing NA-binding and NAI antibodies to N9 have generally been reported at low or undetectable levels [32,51]. Most studies have focused on monoclonal antibody isolation or antibody responses following vaccination rather than pre-existing population immunity [83,84,85]. Notably, a study by Rijal et al. [83] identified a broadly cross-reactive monoclonal antibody (Z2B3) from an H7N9-infected child that inhibited both group 1 N1 and group 2 N9 NAs, which the authors proposed was recalled from pre-existing N1-specific memory B cells induced by prior H1N1 exposure. Although this finding highlights the potential for immune imprinting to generate rare cross-group NAI antibodies, the prevalence and protective significance of cross-reactive immunity against avian N9 viruses at the population level remain unclear.

Collectively, these studies suggest that the extent of population-level cross-reactive NA immunity varies substantially among zoonotic influenza viruses and is largely determined by the antigenic relatedness between seasonal and zoonotic NAs. Human populations possess substantial pre-existing immunity against avian N1 NAs, primarily resulting from exposure to H1N1 viruses, whereas evidence for cross-reactive immunity against other avian NA subtypes remains limited. Understanding the breadth, durability, and determinants of these cross-reactive NA antibody responses will be critical for pandemic risk assessment.

## 6. Future Perspectives and Conclusions

Increasing evidence suggests that population immunity to influenza NA is an important yet underappreciated component of protection against emerging influenza viruses. Seasonal H1N1 virus infection, particularly following the 2009 pandemic, has generated substantial cross-reactive immunity against Eurasian avian N1 neuraminidases circulating in contemporary clade 2.3.4.4b H5N1 viruses. However, this immunity is not uniformly distributed across populations. Birth cohort effects and differences in infection and vaccination histories contribute to substantial age-dependent variation in pre-existing NA antibodies, resulting in potentially heterogeneous susceptibility to emerging influenza viruses. Furthermore, antigenic differences among avian N1 lineages, including Eurasian and North American avian N1 viruses, as well as the continued circulation of genetically distinct H5N1 clades (clade 2.3.2.1a and clade 2.3.2.1e) in Asia, highlight that cross-protection may not extend to all zoonotic viruses.

A key challenge for pandemic preparedness is therefore to develop vaccination strategies that induce broad and durable NA immunity before the emergence of a novel pandemic virus. One promising approach is the development of NA-based vaccines incorporating multiple antigenically distinct NAs or conserved NA antigens, which may broaden the repertoire of NA antibodies capable of recognizing divergent zoonotic viruses. Emerging vaccine platforms, including virus-like particles (VLPs) and mRNA-based approaches, provide opportunities to enhance NA immunogenicity and enable multivalent or structurally optimized NA antigen design [5,86]. However, the effectiveness of such strategies may be influenced by pre-existing immune history and imprinting, which can shape the recruitment and expansion of NA-specific memory B cells. Therefore, identifying broadly inhibitory NA antibodies and defining their conserved epitopes, germline gene usage, and memory B-cell lineages may provide a rational basis for designing vaccines that preferentially direct antibody responses toward conserved regions.

Future studies should also evaluate how NA antigen selection, antigenic breadth, vaccine platform, adjuvant, route of administration, and repeated vaccination influence the magnitude, breadth, durability, and cross-reactivity of NA-specific antibody responses across different age groups and birth cohorts. As zoonotic influenza viruses continue to emerge from avian, swine, and more recently cattle reservoirs, vaccine evaluation should extend beyond homologous NAI titers to assess cross-reactivity against representative avian NAs, including antigenically divergent N1 lineages and other zoonotic NA subtypes. Such assessments will help identify immunity gaps, evaluate population susceptibility, and strengthen pandemic preparedness strategies.

## Data Availability

No new data were created or analyzed in this study. Data sharing is not applicable to this article.

## Abbreviations

The following abbreviations are used in this manuscript:

ADCC	Antibody-dependent cellular cytotoxicity
ADCP	Antibody-dependent cellular phagocytosis
ELISA	Enzyme-linked immunosorbent assay
ELLA	Enzyme-linked lectin assay
HA	Hemagglutinin
HI	Hemagglutination inhibition
HPAI	Highly pathogenic avian influenza
IIV3/IIV4	Trivalent and quadrivalent inactivated influenza vaccines
LAIVs	Live-attenuated influenza vaccines
NA	Neuraminidase
NAI	Neuraminidase inhibition/neuraminidase-inhibiting
NP	Nucleoprotein
M	Matrix gene
MIADA	Multiplex influenza antibody detection assay
MN	Microneutralization
NS	Non-structural gene
PA	Polymerase acidic protein
PB1	Polymerase basic protein 1
PB2	Polymerase basic protein 2
RIVs	Recombinant HA vaccines
VLP	Virus-like particle
